# Soft X-ray Exposure Promotes Na Intercalation in Graphene Grown on Si-Face SiC

**DOI:** 10.3390/ma8084768

**Published:** 2015-07-28

**Authors:** Somsakul Watcharinyanon, Chao Xia, Yuran Niu, Alexei A. Zakharov, Leif I. Johansson, Rositza Yakimova, Chariya Virojanadara

**Affiliations:** 1 Department of Physics, Chemistry, and Biology, Linköping University, Linköping S-58183, Sweden; E-Mails: Somsakul.Watcharinyanon@gknaerospace.com (S.W.); chaxi@ifm.liu.se (C.X.); lij@ifm.liu.se (L.I.J.); roy@ifm.liu.se (R.Y.); 2 MAX-lab, Lund University, Lund S-22100, Sweden; E-Mails: yuran.niu@maxlab.lu.se (Y.N.); alexei.zakharov@maxlab.lu.se (A.A.Z.)

**Keywords:** graphene on Si-face SiC, intercalation of Na, soft X-ray exposure, electron exposure

## Abstract

An investigation of how electron/photon beam exposures affect the intercalation rate of Na deposited on graphene prepared on Si-face SiC is presented. Focused radiation from a storage ring is used for soft X-ray exposures while the electron beam in a low energy electron microscope is utilized for electron exposures. The microscopy and core level spectroscopy data presented clearly show that the effect of soft X-ray exposure is significantly greater than of electron exposure, *i.e.*, it produces a greater increase in the intercalation rate of Na. Heat transfer from the photoelectrons generated during soft X-ray exposure and by the electrons penetrating the sample during electron beam exposure is suggested to increase the local surface temperature and thus the intercalation rate. The estimated electron flux density is 50 times greater for soft X-ray exposure compared to electron exposure, which explains the larger increase in the intercalation rate from soft X-ray exposure. Effects occurring with time only at room temperature are found to be fairly slow, but detectable. The graphene quality, *i.e.*, domain/grain size and homogeneity, was also observed to be an important factor since exposure-induced effects occurred more rapidly on a graphene sample prepared *in situ* compared to on a furnace grown sample.

## 1. Introduction

The effects induced by Na deposited on graphene samples grown on Si-face SiC and kept at room temperature and after subsequent heating have been investigated previously [1,2,3], using photoelectron spectroscopy (PES,ARPES), scanning tunneling microscopy (STM), and low-energy electron microscopy (LEEM). These investigations concluded that partial intercalation in between carbon layers and at the interface occurred directly after deposition, although most of the Na initially remained on the sample surface and droplets of Na also formed. For samples showing a graphene coverage consisting of domains of monolayer and bilayer graphene, respectively, the intercalation was found to be quite inhomogeneous but to occur on both types of domains. The intercalation was shown to begin at boundaries between such domains and also at stripes/streaks on domains with monolayer graphene. Heating at temperatures up to around 100 °C was shown [1,2,3] to strongly promote the intercalation, while heating to higher temperatures resulted in Na de-intercalation and desorption. The intercalation process was also found to be dynamic, *i.e.*, to increase with time but also with electron/photon beam exposure. It is these latter effects that we address in the present investigation, which finds that photon beam exposure produces a significantly more pronounced effect than electron beam exposure and the weak time dependence observed when the sample is kept at room temperature.

Below we report results from LEEM, X-ray photoelectron microscopy (XPEEM), selected area photoelectron spectroscopy (μ-PES) and selected area low energy electron diffraction (μ-LEED) investigations of how electron/photon beam exposures affect intercalation of Na deposited on graphene grown on Si-face SiC. The data presented were collected from a furnace-grown graphene sample [4,5,6]. Data were also collected on a graphene sample grown *in situ.* It showed very similar though more pronounced effects, which we attributed to the considerably smaller graphene domain/grain sizes present on samples grown this way.

## 2. Results and Discussion

**Figure 1:**
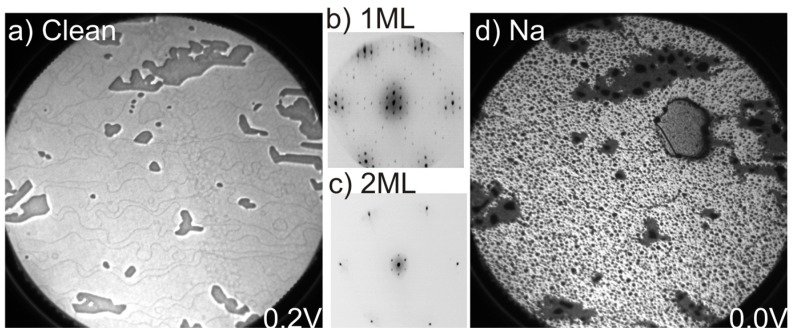
(**a**) LEEM image at a field of view (FOV) of 25 µm and start voltage (SV) of 0.3 eV; μ-LEED pattern from (**b**) 1 to (**c**) 2 ML areas, respectively, of the as-grown sample; and (**d**) LEEM image after Na deposition, SV = 0.0 eV.

The LEEM image in Figure 1a shows a large homogenous monolayer graphene sample at a field of view (FOV) of 25 µm. The dominating bright gray areas represent a coverage of 1 ML graphene while the darker gray areas correspond to 2 ML graphene islands. The number of graphene layers was confirmed by the measured electron reflectivity curves that exhibit a single and double minima corresponding to 1 and 2 ML, respectively [4]. The LEED pattern, in Figure 1b, collected from a 1 ML area shows graphene 1 × 1 spots surrounded by (6√3 × 6√3)*R*30° superstructure spots. The superstructure spots originate from the carbon interface layer, the so-called buffer layer, that forms first upon graphene growth by thermal decomposition of Si-face SiC. This layer forms strong covalent bonds to the SiC substrate and does not exhibit graphitic electronic properties [1,7,8,9]. The graphene layers develop on top of this buffer layer and after intercalation the buffer layer decouples from the substrate and exhibits graphene properties [1,7,8,9]. The LEED pattern collected from a 2 ML area, shown in Figure 1c, show mainly the 1 × 1 graphene spots and weaker buffer layer spots that are suppressed by the additional graphene layer. The LEEM image, in Figure 1d, collected from the same area after Na deposition, with the sample kept at room temperature, reveals quite drastic changes. Dark spots appear all over the surface on both 1 and 2 ML areas but the spots appear to be larger on the 2 ML than on the 1 ML areas. In addition, a large gray droplet with dark edges is observed close to the upper right corner of the image after the deposition. We tentatively interpret this as a bigger Na droplet that has formed on the 1 ML graphene area, and show below using the XPEEM data collected that this is actually the case.

**Figure 2:**
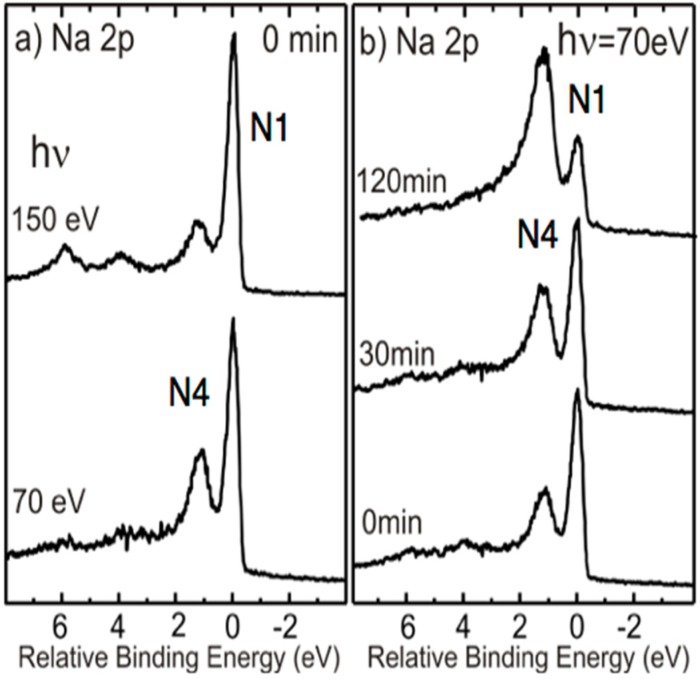
(**a**) Na 2p spectrum recorded directly after Na deposition at two different photon energies; and (**b**) Na 2p spectrum recorded after different times of soft X-ray exposure.

**Figure 3:**
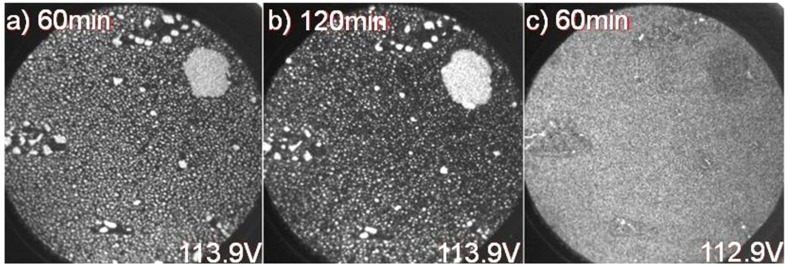
XPEEM images recorded, at a FOV = 25 µm and hv = 150 eV, from the Na 2p component at 0 eV relative binding energy after (**a**) 60 and (**b**) 120 min. of soft X-ray exposure; (**c**) XPEEM image from the other Na 2p component located at around 1.2 eV and after 60 min of soft X-ray exposure.

Na 2p spectra recorded directly after deposition, at two different photon energies, are shown in Figure 2a. The dominant features around 0 and 1.2 eV relative binding energies correspond to components labeled N1 and N4 in the earlier μ-PES study [3] and then assigned to respectively metallic Na clusters/droplets on the surface and to a relatively homogenously adsorbed Na overlayer on the surface. The effects induced in the Na 2p spectrum after exposure to the soft X-ray beam for 30 and 120 min are illustrated in Figure 2b, and show a gradual but pronounced decrease in intensity of the main component, N1, with exposures time while the other component, N4, remains at a fairly constant intensity (note that the spectra in Figure 2b only show relative intensity ratios within each spectrum since they are plotted at a constant maximum peak height). The spatial distribution over the surface of these two components is measured directly in XPEEM when detecting only electrons having a kinetic energy that corresponds to that photoelectron peak. This is illustrated in Figure 3 where (a) and (b) show the intensity variation of photoelectrons from the initially dominating N1 component, originating from Na clusters/droplets on the surface, after 60 and 120 min of soft X-ray beam exposure. In (c) the variation in photoelectron intensity from the higher binding energy N4 component after 60 min of soft X-ray beam exposure is shown and illustrates that it corresponds to a fairly homogenous distribution of Na atoms on the surface. The distribution of clusters/droplets in (a) and (b) is seen to be not so homogenous. The medium sized white spots appear to be concentrated in locations on the surface where boundaries between 1 and 2 ML areas exist.

**Figure 4:**
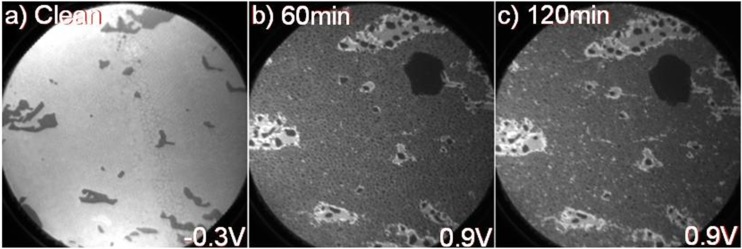
XPEEM image, at a FOV = 25 µm and hv = 150 eV, recorded from (**a**) the as-grown sample at an electron energy of −0.3 eV; and from the sample after Na deposition and subsequent soft X-ray exposure of (**b**) 60 and (**c**) 120 min, respectively, and at an electron energy of 0.9 eV.

The smaller white spots on the other hand appear to be fairly homogenously distributed on 1 ML areas in Figure 3a while in Figure 3b a somewhat more uneven distribution and larger, darker grey areas appear distributed in the 1 ML areas. In the 2 ML areas some medium sized white spots are mainly visible but not the smaller ones. The bigger droplet in the upper right part of the image appears to be about the same size after exposure as before, as judged from a comparison with Figure 1d. The variation in work function over the surface can be monitored by measuring the intensity of emitted secondary electrons near the cut-off energy, *i.e.*, near 0 kinetic energy. This is illustrated for the initial surface in Figure 4a, and shows that the work function is somewhat smaller in the 1 ML graphene areas than in areas with 2 ML. When detecting electrons with a slightly higher kinetic energy, the contrast in the image may originate from additional effects and not only variations in the work function but can nevertheless provide some information about the distribution of the deposited element on a surface. In the XPEEM images in Figure 4b,c, collected after 60 and 120 min of soft X-ray beam exposure, areas with 2 ML graphene appear bright, 1 ML areas light gray, and Na clusters/droplets darker gray. When checking the distribution of darker grey spots in Figure 4b,c more carefully and comparing them with the distribution of white spots in the XPEEM results from the N1 Na 2p component in Figure 3a,b, one finds striking similarities. On the initial 2 ML areas, essentially only-medium sized dark grey (white) spots appear in Figure 4b,c and Figure 3a,b. After 60 min exposure, the distribution of small dark grey (white) spots is fairly homogeneous on the 1 ML areas in Figure 4b (Figure 3a). However, after 120 min exposure, the distribution is less homogenous, which is quite evident in Figure 4c, where more, smaller bright spots are visible on the initial 1 ML areas, which here actually indicates the presence of 2 ML graphene. A gradual intercalation of Na below the carbon buffer layer during soft X-ray exposure would explain the appearance of these bright spots after a prolonged exposure.

**Figure 5:**
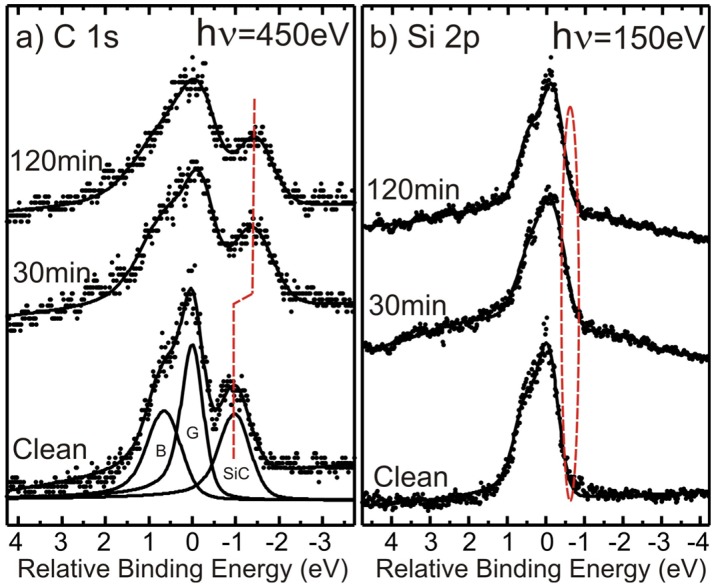
(**a**) C 1s and (**b**) Si 2p spectra recorded from the as prepared sample and after Na deposition and subsequent soft X-ray exposure of 30 and 120 min.

Intercalation of Na below the buffer layer results [1,3] in an increased separation between the graphene and substrate SiC components in the C 1s spectrum and also the appearance of a component shifted to smaller binding energy in the Si 2p spectrum. C 1s and Si 2p core-level spectra recorded before and after 30 and 120 min of soft X-ray beam exposure are shown in Figure 5. The three components required to adequately model the initial C 1s spectrum are displayed in Figure 5a where the binding energy is specified relative to the dominant graphene component (G). The one at lower binding energy originates from the SiC substrate (SiC) and the one at higher binding energy from the carbon buffer layer (B) located on top of the substrate and underneath the graphene. Already, after an exposure of 30 min, the separation between the graphene and substrate components is seen to have increased and after 120 min of exposure the buffer layer component appears to have decreased significantly in relative strength, *i.e.*, no pronounced shoulder is now observable on the high binding energy side of the graphene peak. Both these effects indicate Na intercalation underneath the carbon buffer layer. The effects in the Si 2p spectrum are not so pronounced, but closer inspection reveals that an additional component develops gradually on the low binding energy side of the main peak after soft X-ray exposures. In the μ-PES spectra displayed in Figure 5b, the energy resolution is not so high, so this effect shows up as a broadening on the low energy side of the peak, which is reflected merely by a change in the slope of the low binding energy side of the peak.

**Figure 6:**
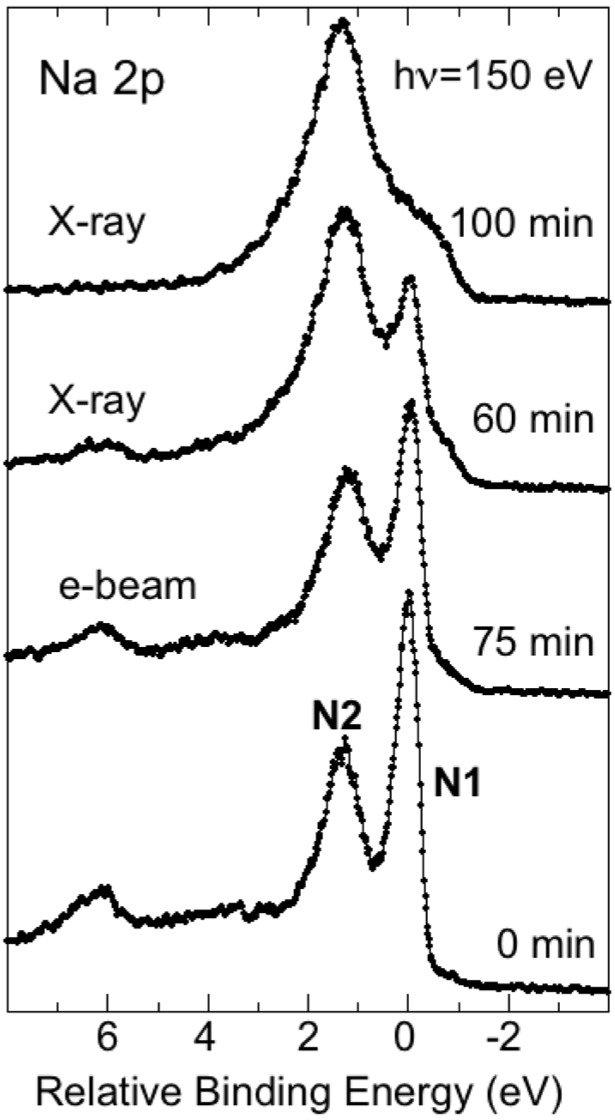
Na 2p spectrum recorded after Na deposition, after 75 min. of exposure to the electron beam and after 60 and 100 min. subsequent exposure to soft X-ray radiation.

In addition to the effects induced by exposures to the soft X-ray radiation beam, we also checked effects over time with the sample in UHV conditions and effects induced by exposures to the electron beam in the LEEM instrument. Exposures to the soft X-ray beam were found to induce the strongest and fastest effects. Leaving the sample overnight in UHV conditions was found to have an effect comparable to about an hour of soft X-ray beam exposure. The effects on a freshly Na-deposited sample from 75 min. exposure to the electron beam in the LEEM followed by exposure to soft X-ray radiation for 60 and 100 min. are illustrated in Figure 6. These spectra illustrate that exposure to the electron beam in the LEEM instrument also affects the deposited Na but to a lesser extent than exposure to the soft X-ray beam. For the purpose of somewhat quantifying these effects, the relative intensity (*i.e.*, the peak area) of the N2 and N1 components was extracted using a peak fit procedure. The N2/N1 intensity ratio is seen to increase with exposure and 75 min. of e-beam exposure is found to increase the ratio from 1.12 to 1.73, meaning an increase by ca. 54% (1.73/1.12 = 1.54). The subsequent soft X-ray exposures of 60 and 100 min. were found to further increase the ratios to, respectively, 3.38 and 6.04. This corresponds to an increase of, respectively, ca. 95% and 241% (3.38/1.73 = 1.95 and 6.04/1.73 = 3.41), and illustrates quite clearly that exposures to soft X-ray induce the strongest and fastest effects.

Similar investigations were repeated on an *in situ* grown graphene sample that had an average graphene coverage of around 1 ML. Similar effects of exposures to soft X-rays were found to appear considerably faster on this sample than on the *ex situ* grown sample. Similar changes to those illustrated in Figure 2 after half an hour of exposure on the *ex situ* sample were observable on this sample after only a few minutes. We attribute this increased rate of Na intercalation on the *in situ* prepared sample to the much higher density of defects, grain boundaries and pits [10] that results on samples prepared this way [9,11], where significantly smaller graphene domain/grain sizes are obtained. This most probably allows for an easier and faster migration of Na atoms through the graphene and carbon buffer layer than on an *ex situ* grown sample.

We can only speculate about why the intercalation is speeded up under soft X-ray and electron beam exposures, since there is no obvious reason why this should happen. We know that the intercalation speed depends greatly on the sample temperature and therefore a plausible reason is an energy–heat transfer from the excited photoelectrons to the lattice, in case of soft X-ray exposure, and from the electrons penetrating the sample in the case of electron beam exposure. These electrons may transfer their energy to optical phonons and thereby increase the local surface temperature. This effect then ought to depend on the electron flux density generated in the exposed area on the sample. In the electron beam exposure, 10 nA illuminated an area of 70 × 80 μm^2^, which gives a flux density of *ca.* 2 pA/μm^2^. These were 5 eV electrons with a fairly narrow energy distribution (*ca.* 0.5 eV). A soft X-ray exposure generates photoelectrons with a wide kinetic energy distribution (from 0 to 150 eV, in principle) so it is not quite correct to compare flux densities only, but it can nevertheless provide a reasonable estimate. Since the electron thermalization time in solids is on the order of picoseconds, the pulse structure of the storage ring has to be taken into account. The MAXII ring has 1 × 10^+8^ bunches per second, each with a pulse duration of about 200 psec. With a photon flux of 1 × 10^+13^ photons/sec, at 150 eV, it means 1 × 10^+5^ photons/pulse(bunch). The number of photoelectrons produced by one photon is specified [12,13] by the total photoionization quantum yield (QY). The QY of our sample that contains mainly C and Si and some Na in the outermost surface layers, is not known, so we have to use an estimate. If we assume a QY of 2% (see motivation below) one photon bunch would create 2000 photoelectrons. At the given pule duration of 200 psec. this would correspond to a photocurrent of 1 × 10^−6^ A. With a photon spot size on the sample of 100 × 100 μm^2^ this corresponds to a an electron flux density of about 100 pA/μm^2^, *i.e.*, 50 times more than in the case of electron beam exposure in the LEEM. This, we suggest, explains the difference in intercalation rates observed between the soft X-ray and electron beam exposures.

How plausible is the estimate of a 2% QY for our sample? For Al, Cu and Au QY values of 5%, 7% and 1%–2% have been reported, respectively [12,13]. Moreover, the total photoelectron yield has been shown [14] to mimic the photoabsorption spectrum, *i.e.*, the absorption coefficient, in the soft X-ray regime. Reported values [15] for the mass absorption coefficient at 150 eV show values of around 1 × 10^5^ cm^2^/g for Si and Al, 5 × 10^4^ for Cu, 1 × 10^4^ for C (graphite) and 6 × 10^3^ for Au. The linear absorption coefficient, defined as the mass absorption coefficient multiplied by the density of the element, then has values of about 4.5, 2.7, 2.3, 1.2 and 0.2 (×10^5^ cm^−1^) for Cu, Al, Si, Au and C, respectively. If we consider that photo-excitation occurs down to a depth determined by the penetration depth of the 150 eV photons, most of that volume contains equal amounts of Si and C. If we then assume that the QY of Si is comparable to that of Al, we arrive at an estimate of a quantum yield of about 2% even if the C atoms do not contribute at all to the quantum yield.

## 3. Experimental Section

Graphene was prepared on the Si-face 4H-SiC substrates by thermal decomposition of SiC in an inductively heated furnace under a highly isothermal condition at a temperature of 2000 °C in an ambient argon pressure of 1 atm [4]. This method is known to produce homogenous, large-area, and high-quality graphene [4,5,6]. The sample used was found to contain mainly one monolayer (1 ML) graphene with a mixture of small 2ML areas/domains. Graphene was also prepared *in situ* by direct heating of a SiC wafer to about 1300 °C for two min., which resulted in a graphene coverage of slightly less than a monolayer, as estimated from recorded C 1s spectra and LEED patterns. Na depositions were performed using a SAES getter source. The sample was kept at room temperature during Na deposition and a coverage of around 2 ML of Na was typically aimed for, and extracted relative Na 2p/Si 2p photoelectron intensities showed the coverage to vary between 1 and 2 ML of Na after the different depositions made. The samples were then exposed either to the soft X-ray beam, the electron beam in the LEEM instrument, or just to ultrahigh vacuum for a specified time. In the soft X-ray exposures, 150 eV photons, with a flux of about 10^13^ photons/sec, were focused onto an area of 100 × 100 μm^2^. In the electron exposures, electrons with a kinetic energy of about 5 eV and a current of 10 nA were focused onto an area of 70 × 80 μm^2^. Changes in the morphology and composition of the sample, and in particular as reflected by the different components appearing in the Na 2p spectrum, were then investigated *versus* exposure time.

The characterizations were performed using an Elmitec spectroscopic photoemission and low energy electron microscope (SPELEEM III) (Elmitec, Claustahl-Zellerfeld, Germany) at beamline I311 at the MAX-lab synchrotron radiation facility in Lund, Sweden. Beamline I311 is equipped with a modified SX700 monochromator, which provides light in the energy range from 43 to 1500 eV. This SPELEEM instrument had a spatial resolution better than 10 nm in the LEEM mode and 30 nm in the PEEM mode. A probing area with a diameter of 10 μm was selected when recording the μ-PES spectra presented above.

## 4. Conclusions

Both electron and photon beam exposures are shown to affect the intercalation rate of Na deposited on graphene, prepared on Si-face SiC. Experimental data collected using LEEM, XPEEM and μ-PES clearly show that the effect of soft X-ray exposures is significantly greater than that of electron exposures, *i.e.*, produces a greater increase in the intercalation rate of Na. Heat transfer from the photoelectrons generated during soft X-ray exposure and by the electrons penetrating the sample during electron beam exposure is suggested to increase the local surface temperature, and thus the intercalation rate. The estimated electron flux density is 50 times greater for soft X-ray exposure compared to electron exposure, which explains the greater increase in the intercalation rate from soft X-ray exposure. The intercalation rate occurring with time only at room temperature and in ultra-high vacuum is observed to be about ten times slower in comparison to soft X-ray exposure. The graphene quality, *i.e.*, graphene domain/grain size and homogeneity, was found to be an important factor since exposure induced effects occurred more rapidly on a graphene sample prepared *in situ* compared to on a furnace grown sample.
